# Modelling data for Predicting New Iron Garnet Thin Films with Perpendicular Magnetic Anisotropy

**DOI:** 10.1016/j.dib.2019.104937

**Published:** 2019-12-06

**Authors:** Saeedeh Mokarian Zanjani, Mehmet C. Onbaşlı

**Affiliations:** a Graduate School of Materials Science and Engineering, Koç University, Sarıyer, 34450, Istanbul, Turkey; b Department of Electrical and Electronics Engineering, Koç University, Sarıyer, 34450, Istanbul, Turkey

**Keywords:** Magnetic anisotropy, Lattice, Substrate, Rare earth iron garnet, Epitaxial

## Abstract

These data include detailed calculations and graphs based on our manuscript submitted to Journal of Magnetism and Magnetic Materials, entitled “Predicting New Iron Garnet Thin Films with Perpendicular Magnetic Anisotropy”. These data are organized in two parts; first, we present the calculated plots of sensitivity of magnetic anisotropy field and anisotropy energy density for 49 epitaxial rare earth iron garnet (REIG) film/substrate pairs (a total of 98 plots, Figs. 1–15). In the second part, we present in Table 1 the complete details on the calculations for total magnetic anisotropy and all material constants used for each of 50 film/substrate pairs. The comparison with the previous experimental demonstrations is also shown in Table 1 (last column) and 2 with an accompanying discussion confirming the reliability of our model.

**Table:** Specifications Table

Subject	Materials Science
Specific subject area	Electronic, optical, and magnetic materials
Type of data	Table Figure Text
How data were acquired	Effective magnetic anisotropy energy density terms and anisotropy field were calculated from ${\text{K}}_{\text{eff}}=-\frac{3}{2}{\text{λ}}_{111}\frac{\text{Y}}{1-\text{v}}{\text{ε}}_{\left|\right|}+2\text{π}{{\text{M}}_{\text{s}}}^{2}+{\text{K}}_{1}$, and ${\text{H}}_{\text{A}}=2\frac{{\text{K}}_{\text{eff}}}{{\text{M}}_{\text{s}}}$ formulae; required parameters for calculations were used from tabulated values, or calculated individually using relevant formulae and filled in Table 1. The plots of Fig. 1, Fig. 2, Fig. 3, Fig. 4, Fig. 5, Fig. 6, Fig. 7, Fig. 8, Fig. 9, Fig. 10, Fig. 11, Fig. 12, Fig. 13, Fig. 14, Fig. 15 were obtained using MATLAB.
Data format	Raw: tabulated intrinsic materials data Analysed: anisotropy terms calculated based on raw data Descriptive: effective magnetic anisotropy behaviour based on analysed data
Parameters for data collection	We used the intrinsic room temperature material properties (bulk saturation magnetization, magnetostriction constants, first-order magnetocrystalline anisotropy K_1_) from experimental references. We used the same Poisson's ratio and Young's moduli for all REIG chemistries in our calculations.
Description of data collection	We collected our raw data from tabulated experimental intrinsic material parameters (lattice parameter, bulk saturation magnetization, Poisson's ratio, Young's modulus, magnetostriction constant, first-order magnetocrystalline anisotropy). Next, we calculated the analysed data (in-plane strain, stress, shape anisotropy K_shape_, magnetoelastic anisotropy K_indu_) using the intrinsic material parameters. Finally, we used our analysed magnetic anisotropy data to calculate the effective anisotropy K_eff_ and its classification as in-plane or perpendicular magnetic anisotropy (PMA).
Data source location	Institution: Koc Univeristy City/Town/Region: Istanbul Country: Turkey.
Data accessibility	Data are presented in this article.
Related research article	Author's name: Saeedeh Mokarian Zanjani, Mehmet Cengiz Onbasli Title: Predicting New Iron Garnet Thin Films with Perpendicular Magnetic Anisotropy Journal: Journal of Magnetism and Magnetic Materials DOI: https://doi.org/10.1016/j.jmmm.2019.166108

**Table:** 

**Value of the Data** • The development of magnetic iron garnets with perpendicular magnetic easy axis (PMA) has been a major materials research area, which enabled researchers to start expanding the physics of spintronics and spin wave devices. • Spintronic devices, especially emerging spin-orbit torque memory and logic devices, are expected to benefit from the development of rare earth iron garnets with tunable magnetic properties, magnetic anisotropy, crystal strain and structure and magnetooptical properties. • There is no previous research in the literature that systematically investigates the ways in which one can change the composition of rare earth iron garnet thin films to tune magnetic anisotropy and achieve room temperature PMA. • The PMA rare earth iron garnet films presented in this article are expected to be of interest for materials scientists working on magnetic oxides and devices, spintronic device researchers working on spin Seebeck effect, spin wave devices, spin logic, spin-orbit torques, all-optical switching, current-controlled magnetism, tunneling magnetoresistance studies, tunnel junctions and other spintronic effects involving unique transport and magnetooptical properties of thin film garnets. • These predicted films offer materials scientists multiple material options to test under a variety of growth and post-processing conditions. This article will be of interest also for spintronics, complex oxide, magnetooptics, spin logic and magnetism researchers.

## 1 Data

**Fig. 1:**
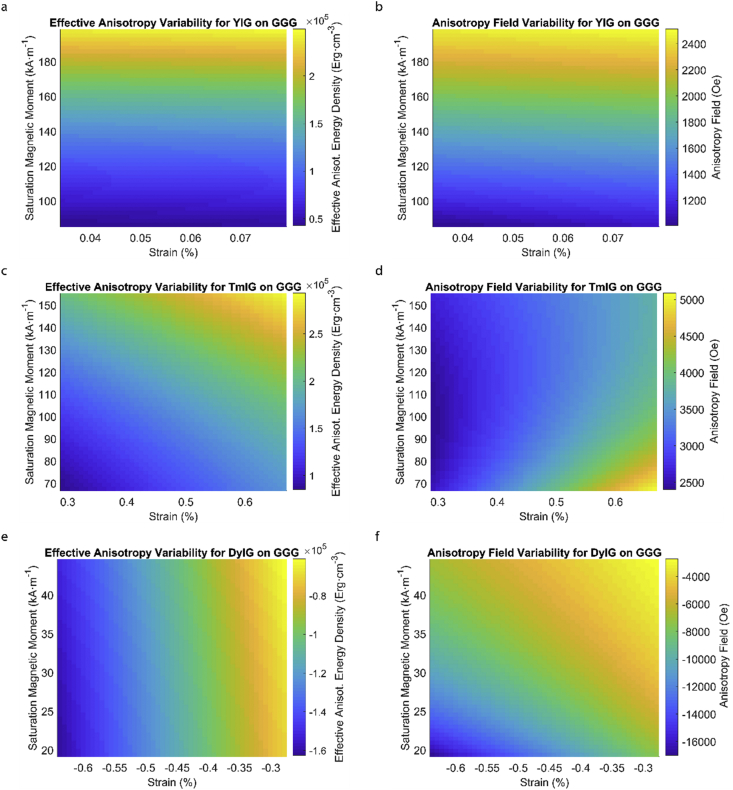
Effect of partial film relaxation or additional strain and saturation magnetic moment variability on the film effective anisotropy energy density and anisotropy field. Variation of effective magnetic anisotropy energy density and anisotropy field, respectively, for (a) and (b) YIG, (c) and (d) TmIG, (e) and (f) DyIG grown on GGG substrate.

**Fig. 2:**
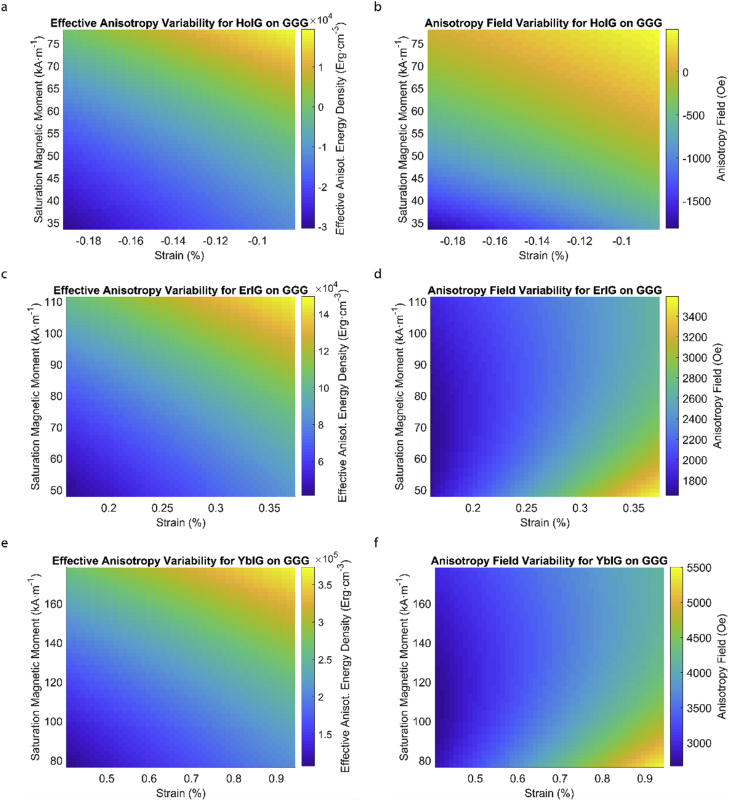
Effect of partial film relaxation or additional strain and saturation magnetic moment variability on the film effective anisotropy energy density and anisotropy field. Variation of effective magnetic anisotropy energy density and anisotropy field, respectively, for (a) and (b) HoIG, (c) and (d) ErIG, (e) and (f) YbIG grown on GGG substrate.

**Fig. 3:**
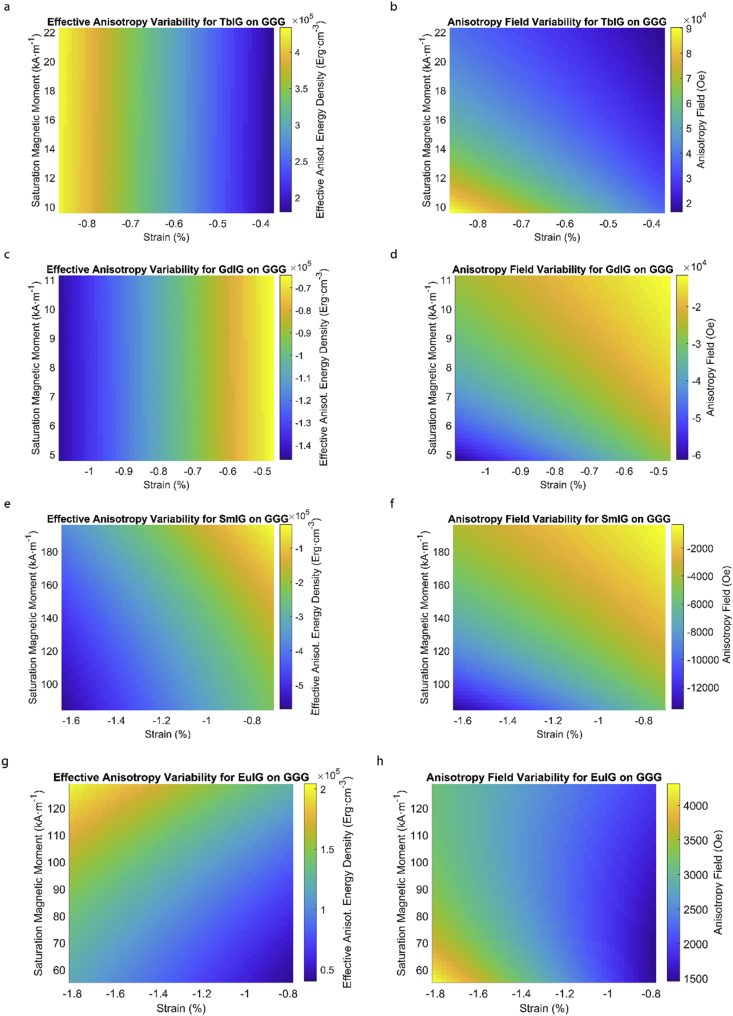
Effect of partial film relaxation or additional strain and saturation magnetic moment variability on the film effective anisotropy energy density and anisotropy field. Variation of effective magnetic anisotropy energy density and anisotropy field, respectively, for (a) and (b) TbIG, (c) and (d) GdIG, (e) and (f) SmIG, (g) and (h) EuIG grown on GGG substrate.

**Fig. 4:**
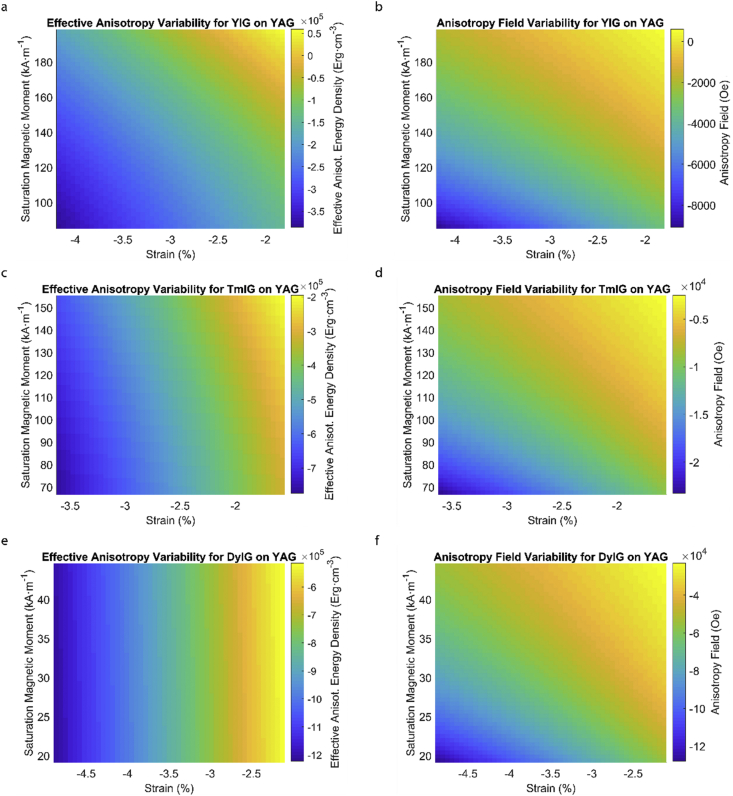
Effect of partial film relaxation or additional strain and saturation magnetic moment variability on the film effective anisotropy energy density and anisotropy field. Variation of effective magnetic anisotropy energy density and anisotropy field, respectively, for (a) and (b) YIG, (c) and (d) TmIG, (e) and (f) DyIG grown on YAG substrate.

**Fig. 5:**
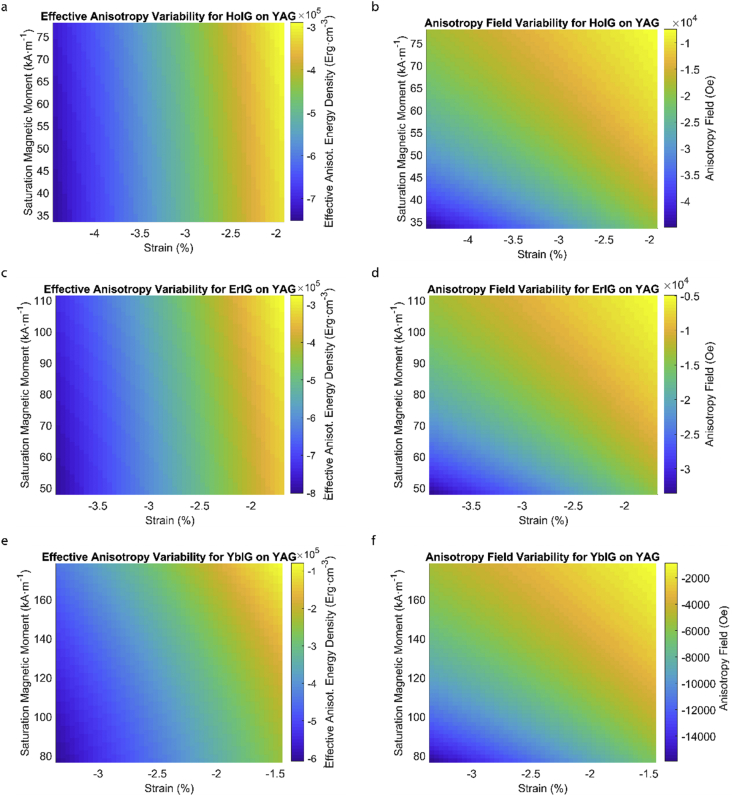
Effect of partial film relaxation or additional strain and saturation magnetic moment variability on the film effective anisotropy energy density and anisotropy field. Variation of effective magnetic anisotropy energy density and anisotropy field, respectively, for (a) and (b) HoIG, (c) and (d) ErIG, (e) and (f) YbIG grown on YAG substrate.

**Fig. 6:**
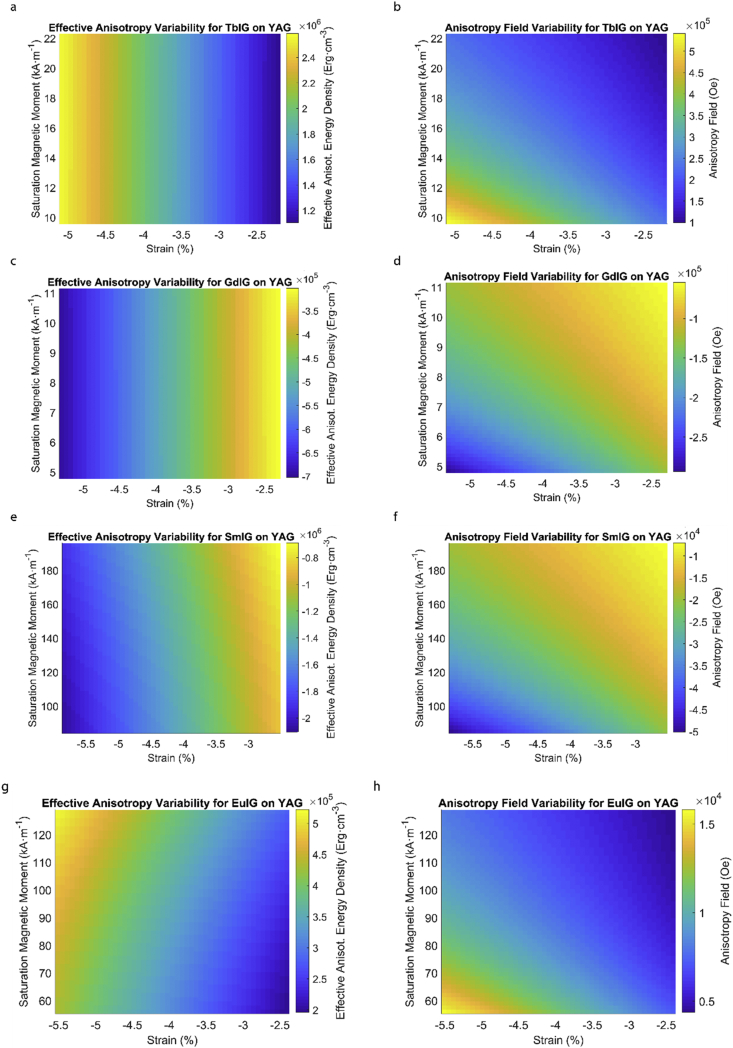
Effect of partial film relaxation or additional strain and saturation magnetic moment variability on the film effective anisotropy energy density and anisotropy field. Variation of effective magnetic anisotropy energy density and anisotropy field, respectively, for (a) and (b) TbIG, (c) and (d) GdIG, (e) and (f) SmIG, (g) and (h) EuIG grown on YAG substrate.

**Fig. 7:**
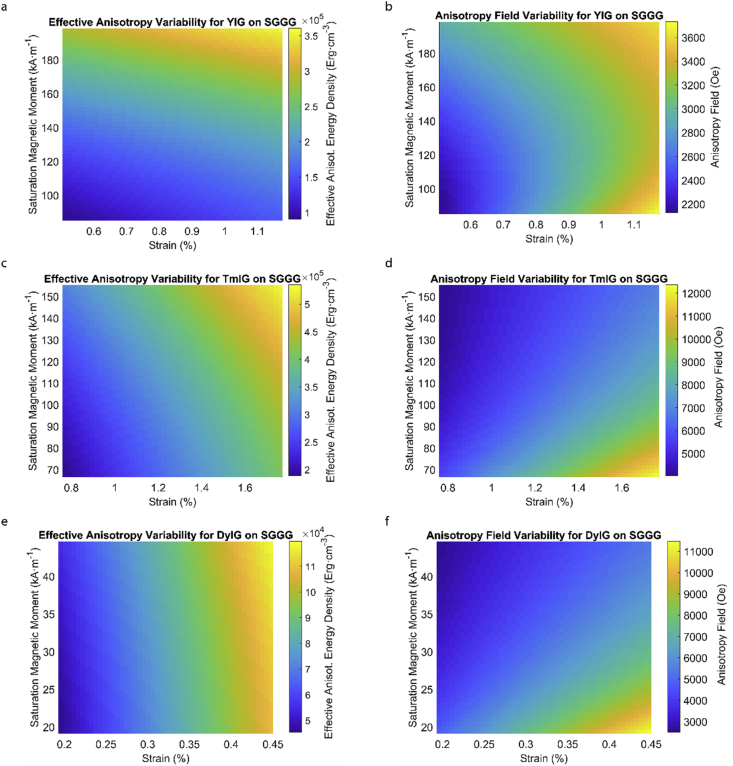
Effect of partial film relaxation or additional strain and saturation magnetic moment variability on the film effective anisotropy energy density and anisotropy field. Variation of effective magnetic anisotropy energy density and anisotropy field, respectively, for (a) and (b) YIG, (c) and (d) TmIG, (e) and (f) DyIG grown on SGGG substrate.

**Fig. 8:**
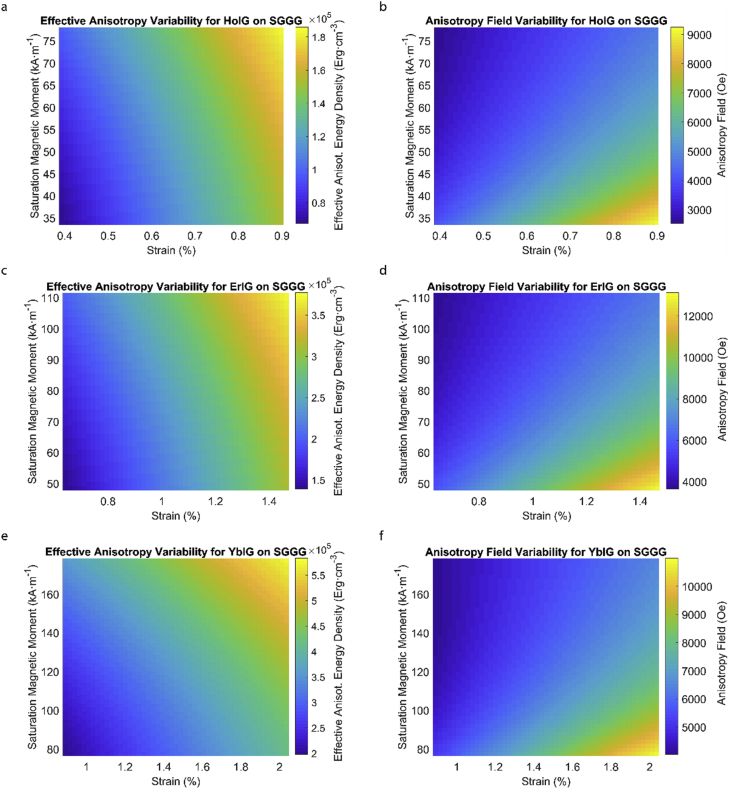
Effect of partial film relaxation or additional strain and saturation magnetic moment variability on the film effective anisotropy energy density and anisotropy field. Variation of effective magnetic anisotropy energy density and anisotropy field, respectively, for (a) and (b) HoIG, (c) and (d) ErIG, (e) and (f) YbIG grown on SGGG substrate.

**Fig. 9:**
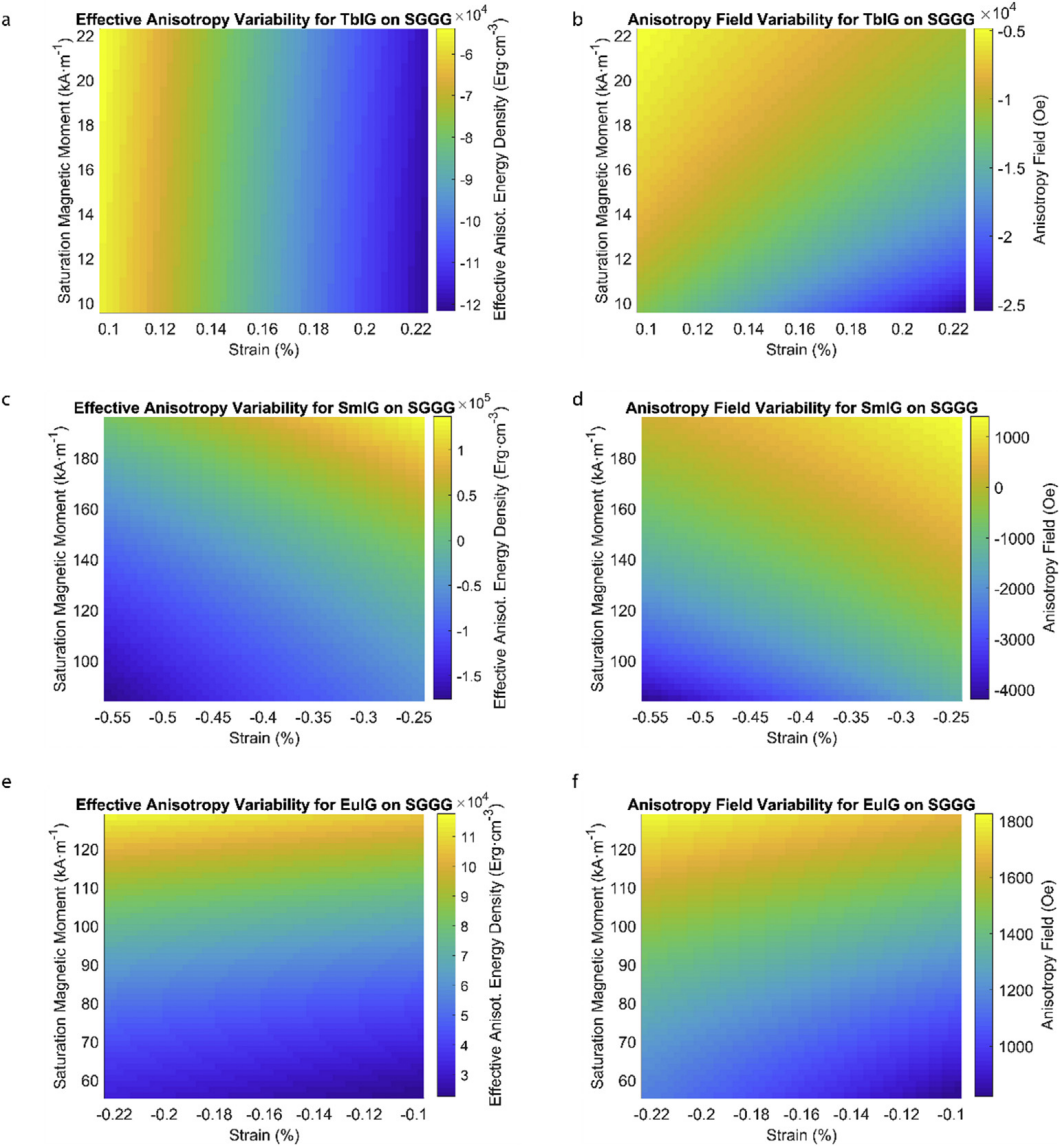
Effect of partial film relaxation or additional strain and saturation magnetic moment variability on the film effective anisotropy energy density and anisotropy field. Variation of effective magnetic anisotropy energy density and anisotropy field, respectively, for (a) and (b) TbIG, (c) and (d) SmIG, (e) and (f) EuIG grown on SGGG substrate.

**Fig. 10:**
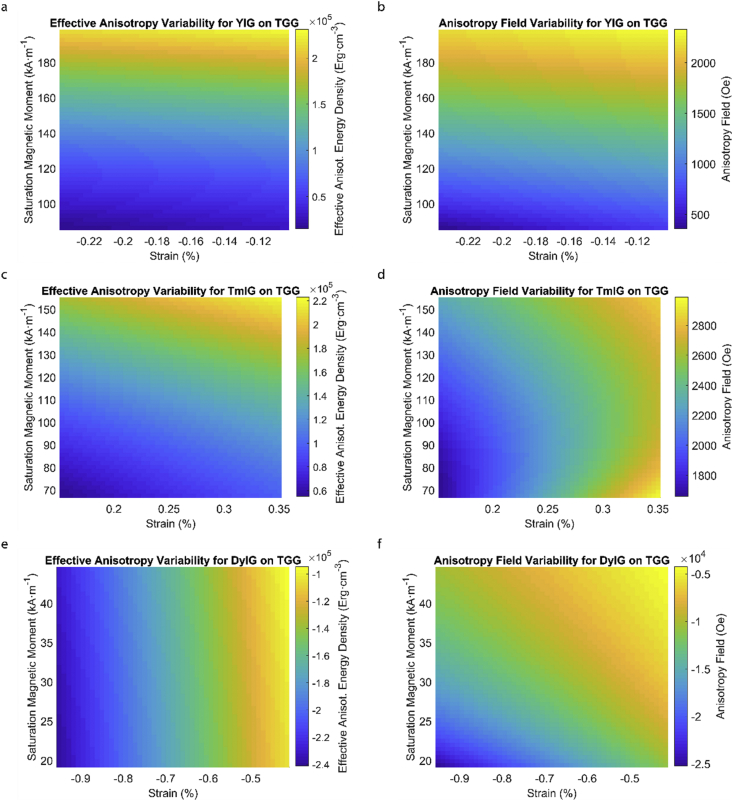
Effect of partial film relaxation or additional strain and saturation magnetic moment variability on the film effective anisotropy energy density and anisotropy field. Variation of effective magnetic anisotropy energy density and anisotropy field, respectively, for (a) and (b) YIG, (c) and (d) TmIG, (e) and (f) DyIG grown on TGG substrate.

**Fig. 11:**
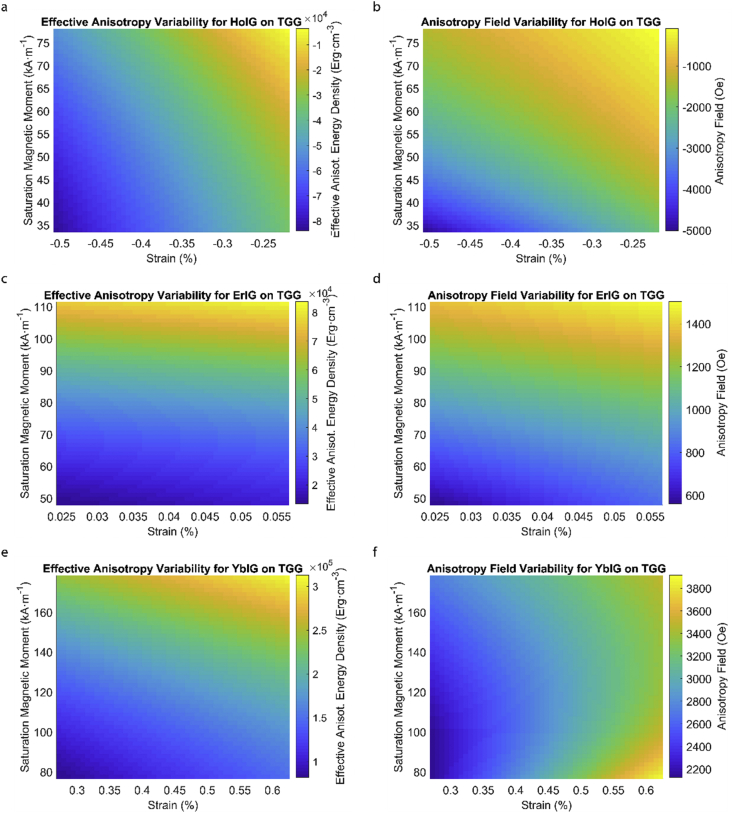
Effect of partial film relaxation or additional strain and saturation magnetic moment variability on the film effective anisotropy energy density and anisotropy field. Variation of effective magnetic anisotropy energy density and anisotropy field, respectively, for (a) and (b) HoIG, (c) and (d) ErIG, (e) and (f) YbIG grown on TGG substrate.

**Fig. 12:**
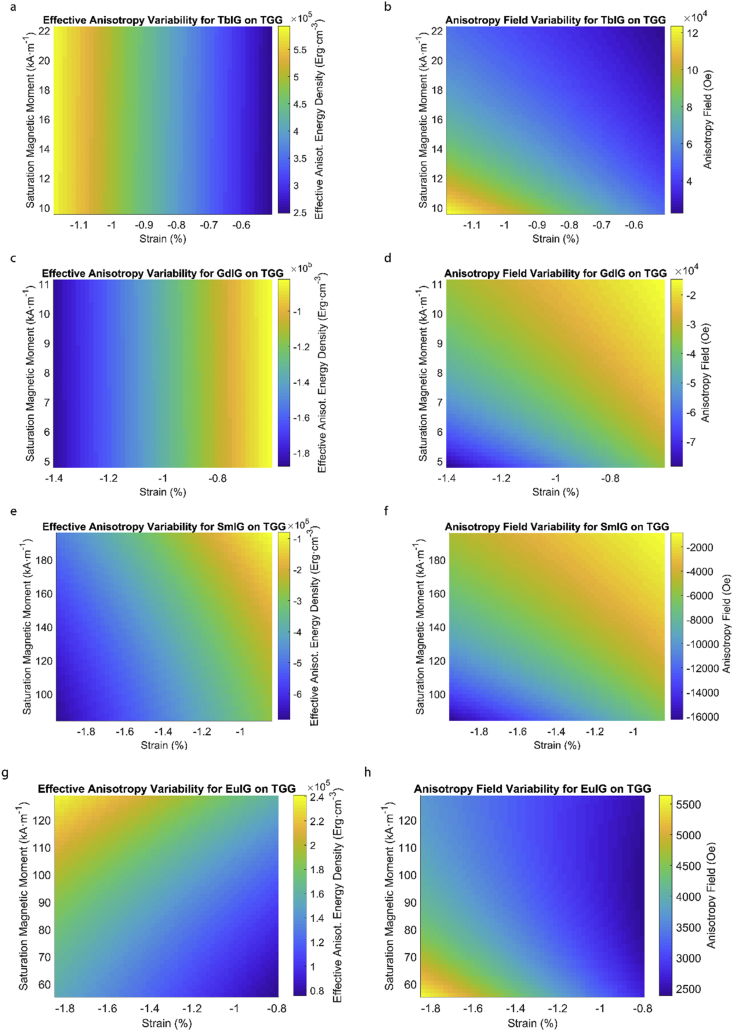
Effect of partial film relaxation or additional strain and saturation magnetic moment variability on the film effective anisotropy energy density and anisotropy field. Variation of effective magnetic anisotropy energy density and anisotropy field, respectively, for (a) and (b) TbIG, (c) and (d) GdIG, (e) and (f) SmIG, (g) and (h) EuIG grown on TGG substrate.

**Fig. 13:**
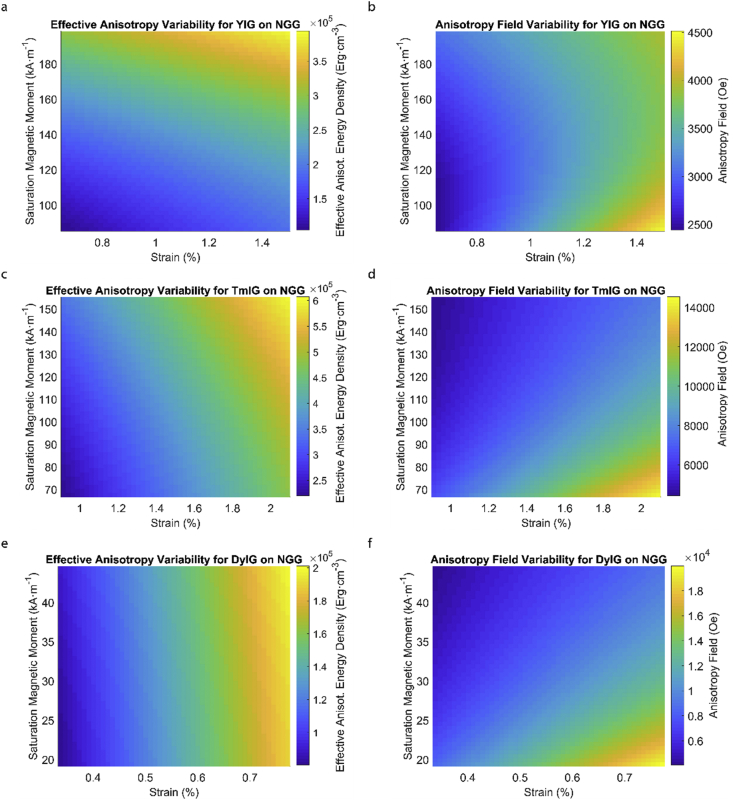
Effect of partial film relaxation or additional strain and saturation magnetic moment variability on the film effective anisotropy energy density and anisotropy field. Variation of effective magnetic anisotropy energy density and anisotropy field, respectively, for (a) and (b) YIG, (c) and (d) TmIG, (e) and (f) DyIG grown on NGG substrate.

**Fig. 14:**
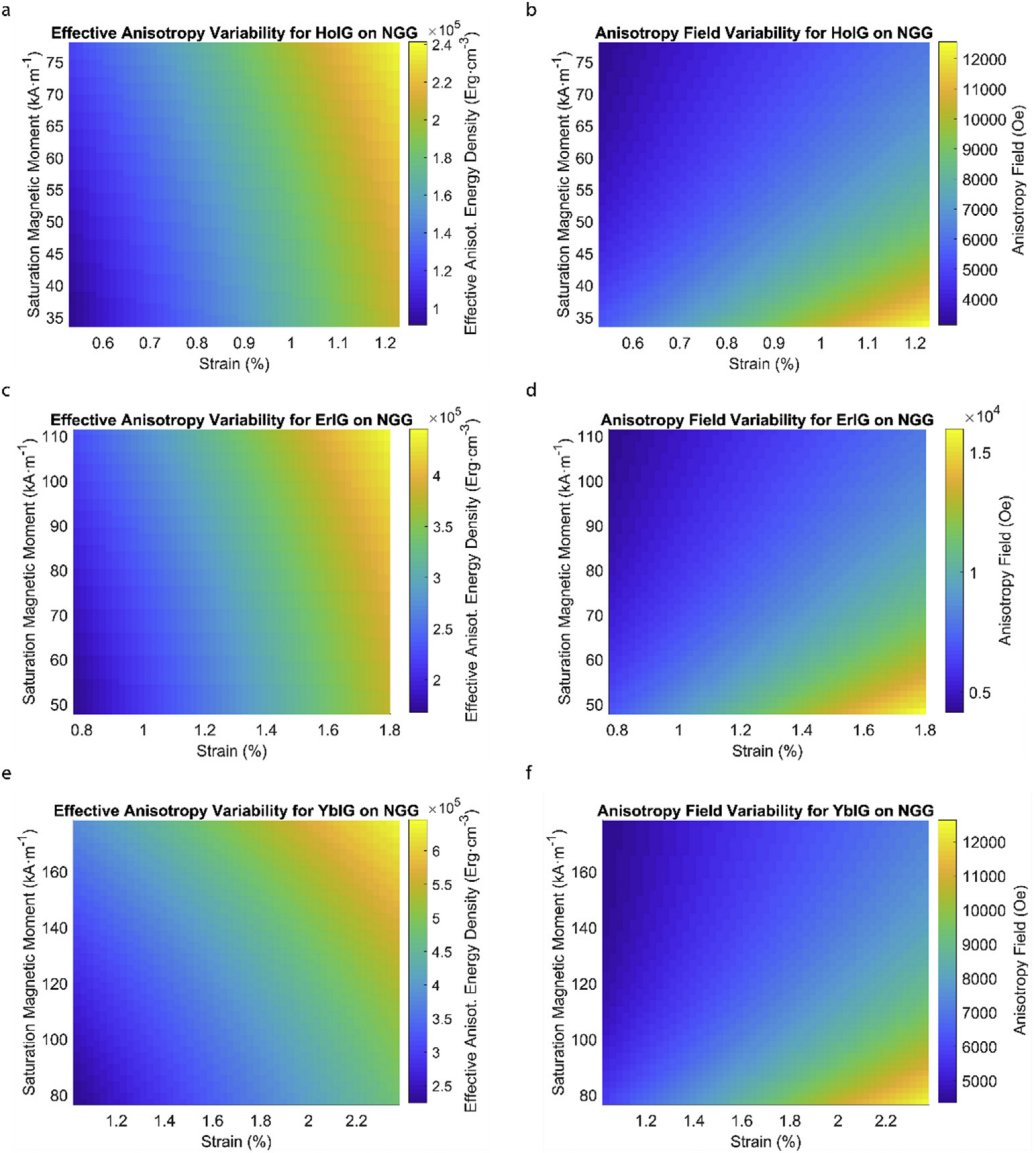
Effect of partial film relaxation or additional strain and saturation magnetic moment variability on the film effective anisotropy energy density and anisotropy field. Variation of effective magnetic anisotropy energy density and anisotropy field, respectively, for (a) and (b) HoIG, (c) and (d) ErIG, (e) and (f) YbIG grown on NGG substrate.

**Fig. 15:**
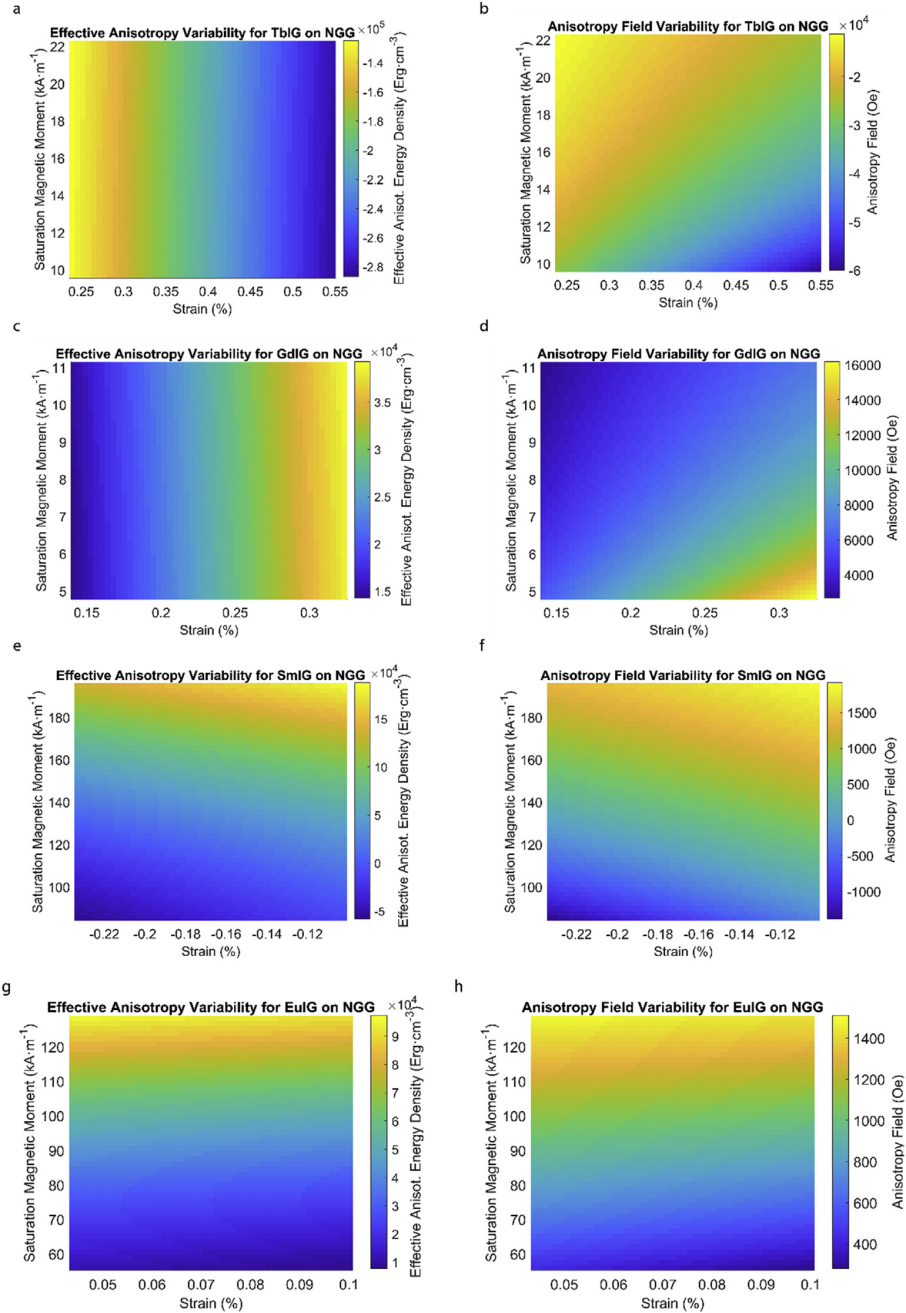
Effect of partial film relaxation or additional strain and saturation magnetic moment variability on the film effective anisotropy energy density and anisotropy field. Variation of effective magnetic anisotropy energy density and anisotropy field, respectively, for (a) and (b) TbIG, (c) and (d) GdIG, (e) and (f) SmIG, (g) and (h) EuIG grown on NGG substrate.

This data article provides a detailed calculation of effective magnetic anisotropy energy density of 50 different rare earth iron garnet/substrate pairs. Fig. 1, Fig. 2, Fig. 3, Fig. 4, Fig. 5, Fig. 6, Fig. 7, Fig. 8, Fig. 9, Fig. 10, Fig. 11, Fig. 12, Fig. 13, Fig. 14, Fig. 15 demonstrate the sensitivity of total magnetic anisotropy energy density (left column) and magnetic anisotropy field (right column) on strain and saturation magnetization variabilities. Variation of effective magnetic anisotropy energy density and anisotropy field, respectively, are shown for Fig. 1. (a) and (b) YIG, (c) and (d) TmIG, (e) and (f) DyIG, Fig. 2. (a) and (b) HoIG, (c) and (d) ErIG, (e) and (f) YbIG, and Fig. 3. (a) and (b) TbIG, (c) and (d) GdIG, (e) and (f) SmIG, (g) and (h) EuIG grown on GGG substrate. In addition, Fig. 4(a–f), Fig. 5(a–f) and Fig. 6(a–h) show the change of K_eff_ and H_a_ with M_s_ and strain of YIG, TmIG, DyIG, HoIG, ErIG, YbIG, TbIG, GdIG, SmIG, and EuIG thin films grown on YAG. Effect of partial film relaxation or additional strain and saturation magnetic moment variability on the film effective anisotropy energy density and anisotropy field of YIG, TmIG, DyIG, HoIG, ErIG, YbIG, TbIG, GdIG, SmIG, and EuIG thin films on SGGG substrate are shown in Fig. 7, Fig. 8, Fig. 9, respectively. Fig. 10, Fig. 11, Fig. 12, Fig. 13, Fig. 14, Fig. 15, demonstrate the variation of K_eff_ and H_a_ with M_s_ and in-plain strain for REIG thin films grown on TGG (Fig. 10, Fig. 11, Fig. 12) and NGG (Fig. 13, Fig. 14, Fig. 15) substrates, respectively. Table 1 shows the theoretical, measured and calculated parameters of effective magnetic anisotropy energy density (K_eff_). Table 2 includes the comparison of magnetic anisotropy state predicted by our model with previous experimental demonstrations.

**Table 1:** Calculation of contributing terms to effective magnetic anisotropy energy density (K_eff_).

RIG	M_s_ (kA·m^−1^)	K_shape_ (erg·cm^−3^ =10^−1^ J·m^−3^)	a_f_ (Å)	ε_||_	σ_||_ (dyn·cm^−2^ =10^−1^ N·m^−2^)	λ_111_	K_indu_ (erg·cm^−3^ = 10^−1^ J·m^−3^)	K_1_ (300K) (erg·cm^−3^ = 10^−1^ J·m^−3^)	K_eff_ (erg·cm^−3^ = 10^−1^ J·m^−3^)	Experimental Demonstration
**a**_**GGG**_**=12.383 Å**										
YIG	141.7	1.26 × 10^5^	12.376	5.66 × 10^−4^	1.59 × 10^9^	−2.40 × 10^−6^	5.74 × 10^3^	−6.10 × 10^3^	1.26 × 10^5^	[7] (Bi-Doped)
TmIG	110.9	7.72 × 10^4^	12.324	4.79 × 10^−3^	1.35 × 10^10^	−5.20 × 10^−6^	1.05 × 10^3^	−5.80 × 10^3^	1.77 × 10^5^	
DyIG	31.8	6.37 × 10^3^	12.44	−4.58 × 10^−3^	−1.29 × 10^10^	−5.90 × 10^−6^	−1.14E × 10^5^	−5.00 × 10^3^	−1.13 × 10^5^	[8] (doped stoichiometry)
HoIG	55.7	1.95 × 10^4^	12.4	−1.37 × 10^−3^	−3.86 × 10^9^	−4.00 × 10^−6^	−2.32 × 10^4^	−5.00 × 10^3^	−8.66 × 10^3^	[9]
ErIG	79.6	3.98 × 10^4^	12.35	2.67 × 10^−3^	7.53 × 10^9^	−4.90 × 10^−6^	5.53 × 10^4^	−6.00 × 10^3^	8.91 × 10^4^	
YbIG	127.4	1.02 × 10^5^	12.3	6.75 × 10^−3^	1.90 × 10^10^	−4.50 × 10^−6^	1.28 × 10^5^	−6.10 × 10^3^	2.24 × 10^5^	
TbIG	15.9	1.59 × 10^3^	12.46	−6.18 × 10^−3^	−1.74 × 10^10^	1.20 × 10^−5^	3.13 × 10^5^	−8.20 × 10^3^	3.07 × 10^5^	
GdIG	7.9	3.98 × 10^2^	12.48	−7.77 × 10^−3^	−2.19 × 10^10^	−3.10 × 10^−6^	−1.02 × 10^5^	−4.10 × 10^3^	−1.06 × 10^5^	[10]
SmIG	140	1.23 × 10^5^	12.53	−1.17 × 10^−2^	−3.30 × 10^10^	−8.60 × 10^−6^	−4.26 × 10^5^	−1.74 × 10^4^	−3.21 × 10^5^	[11]
EuIG	92.1	5.33 × 10^4^	12.5	−1.30 × 10^−2^	−3.65 × 10^10^	1.80 × 10^−6^	9.86 × 10^4^	−3.80 × 10^4^	1.14 × 10^5^	[12] (PMA on GGG (001))
**a**_**YAG**_**=12.005 Å**										
YIG	141.7	1.26 × 10^5^	12.376	−3.00 × 10^−2^	−8.44 × 10^10^	−2.40 × 10^−6^	−3.04 × 10^5^	−6.10 × 10^3^	−1.84 × 10^5^	
TmIG	110.9	7.72 × 10^4^	12.324	−2.59 × 10^−2^	−7.29 × 10^10^	−5.20 × 10^−6^	−5.69 × 10^5^	−5.80 × 10^3^	−4.97 × 10^5^	
DyIG	31.8	6.37 × 10^3^	12.44	−3.50 × 10^−2^	−9.85 × 10^10^	−5.90 × 10^−6^	−8.72 × 10^5^	−5.00 × 10^3^	−8.70 × 10^5^	
HoIG	55.7	1.95 × 10^4^	12.4	−3.19 × 10^−2^	−8.97 × 10^10^	−4.00 × 10^−6^	−5.38 × 10^5^	−5.00 × 10^3^	−5.24 × 10^5^	
ErIG	79.6	3.98 × 10^4^	12.35	−2.79 × 10^−2^	−7.87 × 10^10^	−4.90 × 10^−6^	−5.78 × 10^5^	−6.00 × 10^3^	−5.45 × 10^5^	
YbIG	127.4	1.02 × 10^5^	12.3	−2.40 × 10^−2^	−6.76 × 10^10^	−4.50 × 10^−6^	−4.56 × 10^5^	−6.10 × 10^3^	−3.60 × 10^5^	
TbIG	15.9	1.59 × 10^3^	12.46	−3.65 × 10^−2^	−1.03 × 10^11^	1.20 × 10^−5^	1.85 × 10^6^	−8.20 × 10^3^	1.84 × 10^6^	
GdIG	7.9	3.98 × 10^2^	12.48	−3.81 × 10^−2^	−1.07 × 10^11^	−3.10 × 10^−6^	−4.99 × 10^5^	−4.10 × 10^3^	−5.02 × 10^5^	
SmIG	140	1.23 × 10^5^	12.53	−4.19 × 10^−2^	−1.18 × 10^11^	−8.60 × 10^−6^	−1.52 × 10^6^	−1.74 × 10^4^	−1.42 × 10^6^	
EuIG	92.1	5.33 × 10^4^	12.5	−3.96 × 10^−2^	−1.12 × 10^11^	1.80 × 10^−6^	3.01 × 10^5^	−3.80 × 10^3^	3.51 × 10^5^	
**a**_**SGGG**_**=12.48 Å**										
YIG	141.72	1.26 × 10^5^	12.376	8.40 × 10^−3^	2.37 × 10^10^	−2.40 × 10^−6^	8.52 × 10^4^	−6.10 × 10^3^	2.05 × 10^5^	[7,13] (Bi-Doped)
TmIG	110.908	7.72 × 10^4^	12.324	1.27 × 10^−2^	3.57 × 10^10^	−5.20 × 10^−6^	2.78 × 10^5^	−5.80 × 10^3^	3.50 × 10^5^	[14]
DyIG	31.847	6.37 × 10^3^	12.44	3.22 × 10^−3^	9.06 × 10^9^	−5.90 × 10^−6^	8.02 × 10^4^	−5.00 × 10^3^	8.15 × 10^4^	
HoIG	55.732	1.95 × 10^4^	12.4	6.45 × 10^−3^	1.82 × 10^10^	−4.00 × 10^−6^	1.09 × 10^5^	−5.00 × 10^3^	1.24 × 10^5^	
ErIG	79.618	3.98 × 10^4^	12.35	1.05 × 10^−2^	2.97 × 10^10^	−4.90 × 10^−6^	2.18 × 10^5^	−6.00 × 10^3^	2.52 × 10^5^	
YbIG	127.389	1.02 × 10^5^	12.3	1.46 × 10^−2^	4.12 × 10^10^	−4.50 × 10^−6^	2.78 × 10^5^	−6.10 × 10^3^	3.74 × 10^5^	
TbIG	15.924	1.59 × 10^3^	12.46	1.61 × 10^−3^	4.52 × 10^9^	1.20 × 10^−5^	−8.14 × 10^4^	−8.20 × 10^3^	−8.80 × 10^5^	[15]
GdIG	7.962	3.98 × 10^2^	12.48	0	0	−3.10 × 10^−6^	0	−4.10 × 10^3^	−3.70 × 10^3^	
SmIG	140	1.23 × 10^5^	12.53	−3.99 × 10^−3^	−1.12 × 10^10^	−8.60 × 10^−6^	−1.45 × 10^5^	−1.74 × 10^4^	−3.93 × 10^4^	
EuIG	92.1	5.33 × 10^4^	12.5	−1.60 × 10^−3^	−4.51 × 10^9^	1.80 × 10^−6^	1.22 × 10^4^	−3.80 × 10^3^	6.16 × 10^4^	
**a**_**TGG**_**=12.355 Å**										
YIG	141.72	1.26 × 10^5^	12.376	−1.70 × 10^−3^	−4.78 × 10^9^	−2.40 × 10^−6^	−1.72 × 10^4^	−6.10 × 10^3^	1.03 × 10^5^	
TmIG	110.908	7.72 × 10^4^	12.324	2.52 × 10^−3^	7.09 × 10^9^	−5.20 × 10^−6^	5.53 × 10^4^	−5.80 × 10^3^	1.27 × 10^5^	
DyIG	31.847	6.37 × 10^3^	12.44	−6.83 × 10^−3^	−1.92 × 10^10^	−5.90 × 10^−6^	−1.70 × 10^5^	−5.00 × 10^3^	−1.69 × 10^5^	
HoIG	55.732	1.95 × 10^4^	12.4	−3.63 × 10^−3^	−1.02 × 10^10^	−4.00 × 10^−6^	−6.13 × 10^4^	−5.00 × 10^3^	−4.68 × 10^4^	
ErIG	79.618	3.98 × 10^4^	12.35	4.05 × 10^−4^	1.14 × 10^9^	−4.90 × 10^−6^	8.38 × 10^3^	−6.00 × 10^3^	4.22 × 10^4^	
YbIG	127.389	1.02 × 10^5^	12.3	4.47 × 10^−3^	1.26 × 10^10^	−4.50 × 10^−6^	8.50 × 10^4^	−6.10 × 10^3^	1.81 × 10^5^	
TbIG	15.924	1.59 × 10^3^	12.46	−8.43 × 10^−3^	−2.37 × 10^10^	1.20 × 10^−5^	4.27 × 10^5^	−8.20 × 10^3^	4.21 × 10^5^	
GdIG	7.962	3.98 × 10^2^	12.48	−1.00 × 10^−2^	−2.82 × 10^10^	−3.10 × 10^−6^	−1.31 × 10^5^	−4.10 × 10^3^	−1.35 × 10^5^	
SmIG	140	1.23 × 10^5^	12.53	−1.40 × 10^−2^	−3.93 × 10^10^	−8.60 × 10^−6^	−5.08 × 10^5^	−1.74 × 10^4^	−4.02 × 10^5^	
EuIG	92.1	5.33 × 10^4^	12.5	−1.32 × 10^−2^	−3.72 × 10^10^	1.80 × 10^−6^	1.00 × 10^5^	−3.80 × 10^3^	1.50 × 10^5^	
**a**_**NGG**_**=12.509 Å**										
YIG	141.72	1.26 × 10^5^	12.376	1.07 × 10^−2^	3.03 × 10^10^	−2.40 × 10^−6^	1.09 × 10^5^	−6.10 × 10^3^	2.29 × 10^5^	[13]
TmIG	110.908	7.72 × 10^4^	12.324	1.50 × 10^−2^	4.23 × 10^10^	−5.20 × 10^−6^	3.30 × 10^5^	−5.80 × 10^3^	4.01 × 10^5^	
DyIG	31.847	6.37 × 10^3^	12.44	5.55 × 10^−3^	1.56 × 10^10^	−5.90 × 10^−6^	1.38 × 10^5^	−5.00 × 10^3^	1.40 × 10^5^	
HoIG	55.732	1.95 × 10^4^	12.4	8.79 × 10^−3^	2.48 × 10^10^	−4.00 × 10^−6^	1.49 × 10^5^	−5.00 × 10^3^	1.63 × 10^5^	
ErIG	79.618	3.98 × 10^4^	12.35	1.29 × 10^−2^	3.63 × 10^10^	−4.90 × 10^−6^	2.67 × 10^5^	−6.00 × 10^3^	3.00 × 10^5^	
YbIG	127.389	1.02 × 10^5^	12.3	1.70 × 10^−2^	4.79 × 10^10^	−4.50 × 10^−6^	3.23 × 10^5^	−6.10 × 10^3^	4.19 × 10^5^	
TbIG	15.924	1.59 × 10^3^	12.46	3.93 × 10^−3^	1.11 × 10^10^	1.20 × 10^−5^	−1.99 × 10^5^	−8.20 × 10^3^	−2.06 × 10^5^	
GdIG	7.962	3.98 × 10^2^	12.48	2.32 × 10^−3^	6.55 × 10^9^	−3.10 × 10^−6^	3.04 × 10^4^	−4.10 × 10^3^	2.67 × 10^4^	
SmIG	140	1.23 × 10^5^	12.53	−1.68 × 10^−3^	−4.72 × 10^9^	−8.60 × 10^−6^	−6.09 × 10^4^	−1.74 × 10^4^	4.48 × 10^4^	
EuIG	92.1	5.33 × 10^4^	12.5	7.20 × 10^−4^	2.03 × 10^9^	1.80 × 10^−6^	−5.48 × 10^3^	−3.80 × 10^3^	4.40 × 10^4^

**Table 2:** Comparison of experimental demonstrations of magnetic anisotropy and our model predictions (IP: in-plane, OP: out-of-plane, NA: Not Available).

No.	Thin Film-Substrate combination	Predicted anisotropy	Published Experimental Studies	Does our prediction match the experiment?	Model does not take into consideration:
1	YIG/GGG	IP	[13]	YES	
2	TmIG/GGG	IP	[16]	NO	Off-stoichiometry
3	DyIG/GGG	OP	[8](doped stoichiometry)	YES	
4	HoIG/GGG	OP	[9]	YES	
5	ErIG/GGG	IP	[17]-not thin film	YES	
6	YbIG/GGG	IP	NA		
7	TbIG/GGG	IP	[15]	NO	Ref. [15] contains significant shear stress and reduced ME anisotropy. Off-stoichiometry changes the assumed M_s_, K_1_ and λ_111_.
8	GdIG/GGG	OP	[10]	YES	
9	SmIG/GGG	OP	[11]	YES	
10	EuIG/GGG	IP	[15]	NO	Ref. [15] contains significant shear stress and reduced ME anisotropy. Off-stoichiometry changes the assumed M_s_, K_1_ and λ_111_.
11	YIG/YAG	OP	NA		
12	TmIG/YAG	OP	NA		
13	DyIG/YAG	OP	NA		
14	HoIG/YAG	OP	NA		
15	ErIG/YAG	OP	NA		
16	YbIG/YAG	OP	NA		
17	TbIG/YAG	IP	NA		
18	GdIG/YAG	OP	NA		
19	SmIG/YAG	OP	NA		
20	EuIG/YAG	IP	NA		
21	YIG/SGGG	IP	[13]	NO	Larger λ_111_, strain and M_s_ used in ref. [13]
22	TmIG/SGGG	IP	[14]	NO	Off-stoichiometry
23	DyIG/SGGG	IP	NA		
24	HoIG/SGGG	IP	NA		
25	ErIG/SGGG	IP	NA		
26	YbIG/SGGG	IP	NA		
27	TbIG/SGGG	OP	[15]	NO	Ref. [15] contains significant shear stress and reduced ME anisotropy. Off-stoichiometry changes the assumed M_s_, K_1_ and λ_111_.
28	GdIG/SGGG	OP	NA		
29	SmIG/SGGG	OP	NA		
30	EuIG/SGGG	IP	NA		
31	YIG/TGG	IP	NA		
32	TmIG/TGG	IP	NA		
33	DyIG/TGG	OP	NA		
34	HoIG/TGG	OP	NA		
35	ErIG/TGG	IP	NA		
36	YbIG/TGG	IP	NA		
37	TbIG/TGG	IP	NA		
38	GdIG/TGG	OP	NA		
39	SmIG/TGG	OP	NA		
40	EuIG/TGG	IP	NA		
41	YIG/NGG	IP	[13]	NO	Larger λ_111_, strain and M_s_ used in ref. [13]
42	TmIG/NGG	IP	NA		
43	DyIG/NGG	IP	NA		
44	HoIG/NGG	IP	NA		
45	ErIG/NGG	IP	NA		
46	YbIG/NGG	IP	NA		
47	TbIG/NGG	OP	NA		
48	GdIG/NGG	IP	NA		
49	SmIG/NGG	IP	NA		
50	EuIG/NGG	IP	NA		

## 2 Experimental design, materials and methods

### 2.1 Analytical calculation method of magnetic anisotropy energy density and field

In order to calculate the effective anisotropy energy density we used K_eff_ = K_indu_ + K_shape_ + K_1_ equation to calculate the total anisotropy energy density for 50 thin film rare earth iron garnet/substrate pairs. Fig. 1, Fig. 2, Fig. 3, Fig. 4, Fig. 5, Fig. 6, Fig. 7, Fig. 8, Fig. 9, Fig. 10, Fig. 11, Fig. 12, Fig. 13, Fig. 14, Fig. 15 exclude the Gadolinium Iron Garnet (GdIG) film on substituted Gadolinium Gallium Garnet (SGGG) substrate because there is no lattice mismatch between the film and the substrate. Each anisotropy term consist of the following parameters: ${\text{K}}_{\text{eff}}=-\frac{3}{2}{\text{λ}}_{111}\frac{\text{Y}}{1-\text{v}}{\text{ε}}_{\left|\right|}+2\text{π}{{\text{M}}_{\text{s}}}^{2}+{\text{K}}_{1}$. The energy density was calculated based on the parameters reported in previous references [[1], [2], [3], [4], [5], [6]] and calculated terms according to their formulae (i.e.${\text{ ε}}_{\left|\right|}=\frac{{\text{a}}_{\text{sub}}-{\text{a}}_{\text{film}}}{{\text{a}}_{\text{film}}}$ First-order magnetocrystalline anisotropy, K_1_, is an intrinsic temperature-dependent constant reported for each REIG material. Young's modulus (Y), Poisson's ratio (ν) and magnetostriction constant (λ_111_) parameters evolving in the magnetoelastic anisotropy energy density term (first term) are considered to be constant according to the values previously reported. For shape anisotropy energy calculations (second term), bulk saturation magnetization (M_s_) for each film was used. Since each film may exhibit variability in M_s_ with respect to bulk, the model presented here yields the most accurate predictions when the experimental film M_s_, λ_111_, Y, ν and K_1_, and in-plane strain values are entered for each term. Table 1 shows the theoretical, measured and calculated parameters of anisotropy energy density terms and contributing parameters. In Table 2, we present a comparison of our model's predictions with the previous experimental studies. Anisotropy fields were calculated using ${\text{H}}_{\text{A}}=2\frac{{\text{K}}_{\text{eff}}}{{\text{M}}_{\text{s}}}$ formula. The original Microsoft Excel and MATLAB files used for generating the data for Fig. 1, Fig. 2, Fig. 3, Fig. 4, Fig. 5, Fig. 6, Fig. 7, Fig. 8, Fig. 9, Fig. 10, Fig. 11, Fig. 12, Fig. 13, Fig. 14, Fig. 15 are also presented.

### 2.2 Predictive capability and validity of our model

We tested the prediction accuracy of our model by going through each available experimental demonstration of garnet thin film/substrate anisotropy characterization and comparing their measured anisotropy with the predictions of our model. Below, we show the prediction accuracy and cases where experiments are different from our predictions.

As shown in the table above, our model is able to predict the magnetic anisotropy state of almost all garnet/substrate combinations.
